# Evaluating Electromyography and Sonomyography Sensor Fusion to Estimate Lower-Limb Kinematics Using Gaussian Process Regression

**DOI:** 10.3389/frobt.2022.716545

**Published:** 2022-03-21

**Authors:** Kaitlin G. Rabe, Nicholas P. Fey

**Affiliations:** ^1^ Department of Biomedical Engineering, The University of Texas at Austin, Austin, TX, United States; ^2^ Texas Robotics Center of Excellence, The University of Texas at Austin, Austin, TX, United States; ^3^ Walker Department of Mechanical Engineering, The University of Texas at Austin, Austin, TX, United States

**Keywords:** electromyography—EMG, ambulation, ultrasound, kinematics, regression

## Abstract

Research on robotic lower-limb assistive devices over the past decade has generated autonomous, multiple degree-of-freedom devices to augment human performance during a variety of scenarios. However, the increase in capabilities of these devices is met with an increase in the complexity of the overall control problem and requirement for an accurate and robust sensing modality for intent recognition. Due to its ability to precede changes in motion, surface electromyography (EMG) is widely studied as a peripheral sensing modality for capturing features of muscle activity as an input for control of powered assistive devices. In order to capture features that contribute to muscle contraction and joint motion beyond muscle activity of superficial muscles, researchers have introduced sonomyography, or real-time dynamic ultrasound imaging of skeletal muscle. However, the ability of these sonomyography features to continuously predict multiple lower-limb joint kinematics during widely varying ambulation tasks, and their potential as an input for powered multiple degree-of-freedom lower-limb assistive devices is unknown. The objective of this research is to evaluate surface EMG and sonomyography, as well as the fusion of features from both sensing modalities, as inputs to Gaussian process regression models for the continuous estimation of hip, knee and ankle angle and velocity during level walking, stair ascent/descent and ramp ascent/descent ambulation. Gaussian process regression is a Bayesian nonlinear regression model that has been introduced as an alternative to musculoskeletal model-based techniques. In this study, time-intensity features of sonomyography on both the anterior and posterior thigh along with time-domain features of surface EMG from eight muscles on the lower-limb were used to train and test subject-dependent and task-invariant Gaussian process regression models for the continuous estimation of hip, knee and ankle motion. Overall, anterior sonomyography sensor fusion with surface EMG significantly improved estimation of hip, knee and ankle motion for all ambulation tasks (level ground, stair and ramp ambulation) in comparison to surface EMG alone. Additionally, anterior sonomyography alone significantly improved errors at the hip and knee for most tasks compared to surface EMG. These findings help inform the implementation and integration of volitional control strategies for robotic assistive technologies.

## Introduction

The continued progression of robotic (i.e., powered) lower-limb assistive devices with enhanced sensing, control and actuation has the ability to improve personalized rehabilitation strategies, restore independence and improve quality of life for individuals with mobility disorders and lower-limb loss. Significant improvements of these devices include reduction in their overall weight, improved energy-transfer capabilities *via* injection and recycling, and the increase in overall number of powered joints (Price et al., 2019). However, when considering their physical human-robot interaction, the increased complexity of these devices, including the number of powered joints and their capabilities, is met with an increase in the degrees-of-freedom of the overall control problem. Therefore, sensing modalities that can accurately detect motion of multiple degrees-of-freedom for precise mapping to multiple joints’ control parameters are critical for device translation and clinical adoption.

A breadth of wearable sensing modalities and control schemes have been explored for the accurate recognition of user movement intent. The primary control schemes include classifier-based control and volitional control, as well as some combination of the two, often referred to as shared or indirect volitional control. Classifier-based control typically identifies the mode of ambulation or specific task (Hargrove et al., 2011; Huang et al., 2011; Young et al., 2014). Conversely, volitional control allows the user to execute voluntary movements by more directly mapping user intent to a device output, such as angle, velocity or torque (Ha et al., 2011; Kannape and Herr, 2014). Recent studies that evaluated online performance of shared volitional (i.e., semi-volitional) control of powered lower-limb prostheses have proven successful for providing additional capabilities to the user, such as crossing over obstacles during level walking (Mendez et al., 2020). However, to enable users the access to the full range of capabilities of powered assistive devices, such as use during weight-bearing and non-weight-bearing, it may be best to enable a more fully-volitional control scheme, and one that incorporates intent recognition of multiple degrees-of-freedom. When there are multiple powered joints, such as knees and ankles in a transfemoral prostheses or both hips and knees in an exoskeleton, volitional control over multiple degrees-of-freedom simultaneously would be ideal.

Traditionally, researchers have relied on mechanical sensors as well as neural sensors for classifier-based control and volitional control techniques. Mechanical sensors are used for extracting joint kinematics and kinetics with great success, and are advantageous for aiding in shared volitional control (Mendez et al., 2020). However, these sensors record kinematic or kinetic signals as the movement occurs and thus do not always precede impending motion. Non-invasive neural sensors, e.g. surface electromyography (EMG), are commonly used to extract muscle activation information that precedes joint movement (Farina et al., 2004). However, surface EMG is susceptible to muscle crosstalk and accesses muscle activity information from superficial muscles (Farina et al., 2002). Furthermore, muscle activity is only one contributing factor to muscle force production and resulting joint motion (Disselhorst-Klug et al., 2009). Still, EMG remains the primary peripheral sensing modality to probe information about muscle contraction that precedes limb motion. Recent advances in high-density recording and motor unit decomposition have enabled an increased signal dimensionality of EMG and understanding of the motor unit activity within superficial muscles (Merletti et al., 2008; Stango et al., 2015). However, alternative sensing modalities could provide additional information about muscle contraction, especially the contraction of multiple muscles that are within close proximity of one another and those that are not superficial. Advances are needed to enable accurate prediction of joint kinematics for multiple degrees-of-freedom during varying forms of ambulation (e.g., level ground, stair and ramp ambulation).

In comparison to EMG, which provides information about muscle activity, dynamic ultrasound imaging of skeletal muscle tissue, i.e. sonomyography, has the ability to capture real-time information about muscle deformation that is related to both fiber length and contractile velocity, two additional contributing factors to muscle force and resulting joint motion (Hallock et al., 2018). Additionally, sonomyography can access information from both superficial and deep muscles, resulting in increased spatial resolution and overall dimensionality of the signal (Rabe et al., 2021a). Multiple researchers have achieved success using sonomyography for control of multiple degrees-of-freedom in the upper limb (Shi et al., 2010; Hettiarachchi et al., 2015; Dhawan et al., 2019). In addition, previous research demonstrated improvement to classification of discrete ambulation tasks by sonomyography compared to surface electromyography (Rabe et al., 2021a). Further, sonomyography of the lower limb has been used to continuously estimate knee kinematics (Jahanandish et al., 2019b; Rabe et al., 2020a), as well as hip, knee and ankle moments during basic walking tasks and isometric contraction (Rabe et al., 2020b; Zhang et al., 2020; Zhang et al., 2021). However, the ability of sonomyography to continuously predict multiple joint kinematics during widely varying ambulation tasks encompassing level ground, ramp incline, ramp decline, stair ascent and stair descent and how the performance compares to surface EMG as well as the fusion of surface EMG with sonomyography is unknown. Identifying these important relationships would help inform the implementation and capabilities of volitional controllers for advanced lower-limb assistive technologies.

The objective of this research is to use both surface EMG and sonomyography, as well as the fusion of features from both sensing modalities, as inputs to a Gaussian process regression model for the continuous estimation of healthy subjects’ hip, knee and ankle angles and angular velocities during five ambulation tasks. Gaussian process regression is a kernel-based Bayesian technique for nonlinear regression that has been introduced as an alternative to musculoskeletal model-based solutions to inverse dynamics. In this study, regression models were trained and tested in task-invariant frameworks such that strides from all ambulation tasks were included in both the training dataset and testing dataset. We hypothesized that sonomyography would improve regression prediction of hip and knee kinematics in comparison to surface EMG, but that sensor fusion of sonomyography with surface EMG would be required for accurate performance of ankle kinematic estimation. We further hypothesized that prediction of joint angle would outperform prediction of joint angular velocity due to the increased noise and variability of the angular velocity signal—noise that is associated with the use of numerical differentiation to calculate this signal from motion capture data. Results from this research can inform strategies for implementing multiple degree-of-freedom control over powered assistive devices using features extracted from novel sensing modalities such as sonomyography and traditional sensing modalities like EMG.

## Materials and Methods

**FIGURE 1:**
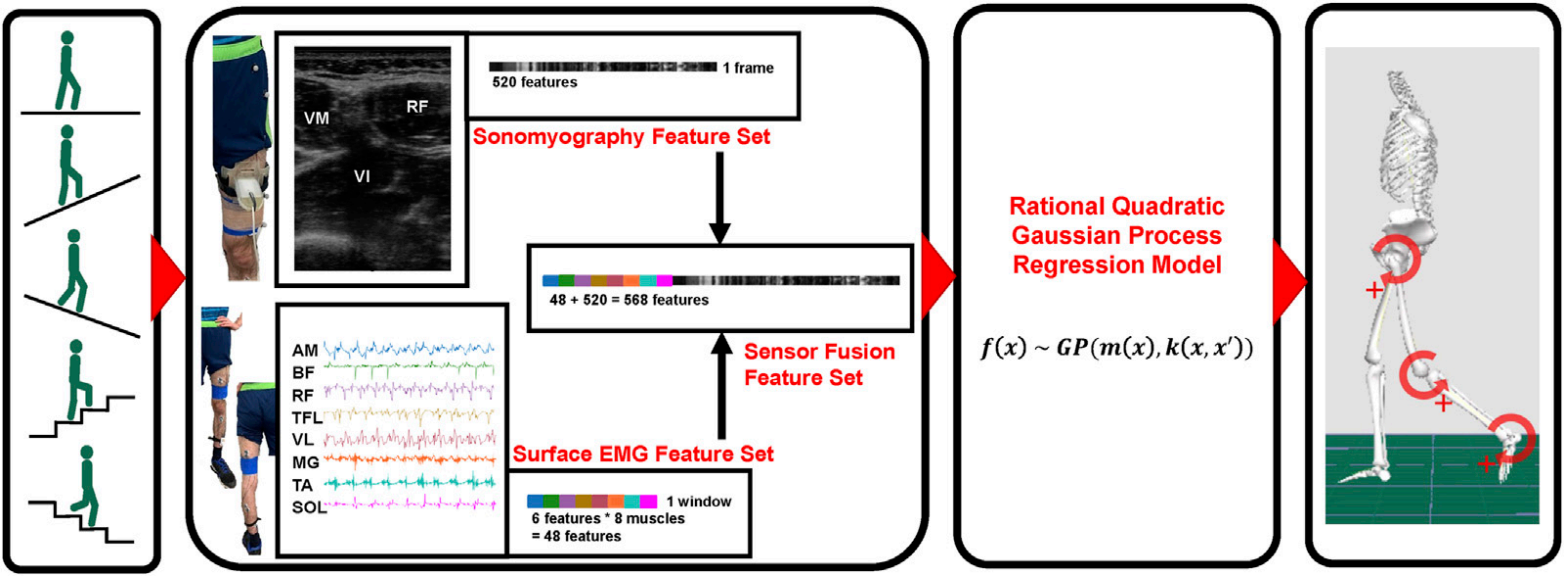
Schematic representation of methods. Beginning with data collection during five ambulation modes, sensing modality [sonomyography, surface electromyography (EMG) and sensor fusion] feature generation, regression model implementation, and ultimate hip, knee and ankle joint kinematic prediction.

A schematic overview of the methods beginning with data collection through model prediction of joint kinematics is given in Figure 1. This study was reviewed and approved by The University of Texas at Dallas institutional review board. The participants provided their written informed consent prior to participating in this study.

### Subjects and Data Collection

Nine able-bodied subjects (five male and four female) were asked to complete two sets of ambulation tasks including: level walk, 10° incline walk, 10° decline walk, stair ascent and stair descent. Additional subject data can be found in Table 1. Prior to two sets of experiments, subjects were equipped with reflective markers for tracking kinematics, surface EMG for recording muscle activity and ultrasound sensors for collection of sonomyography data.

**TABLE 1:** Mean and standard deviation (SD) subject characteristics (*N* = 9).

Subject characteristic	Mean (SD)
Age (years)	29.9 (11.2)
Height (m)	1.72 (0.11)
Weight (kg)	65.8 (10.4)
Anterior Ultrasound Penetration Depth (cm)	6.0 (0.6)
Posterior Ultrasound Penetration Depth (cm)	6.0 (0.3)
Level Walk Speed (m/s)	0.79 (0.15)
Incline Walk Speed (m/s)	0.64 (0.11)
Decline Walk Speed (m/s)	0.62 (0.11)
# of Stair Ascent Strides Included in Analyses	7.8 (2.4)
# of Stair Descent Strides Included in Analyses	9.6 (3.4)

First, pre-gelled, self-adhesive electrodes (H124SG Covidien, Medtronic Inc., Dublin, Ireland) were placed over the belly of eight muscles with an inter-electrode distance of 2 cm. Electrodes were connected to a Bluetooth EMG unit (Shimmer3 EMG Unit, Shimmer, Dublin, Ireland) and EMG signals were recorded from the adductor magnus (AM), biceps femoris (BF), rectus femoris (RF), vastus lateralis (VL), tensor fascia latae (TFL), medial gastrocnemius (MG), tibialis anterior (TA) and soleus (SOL) muscles at a frequency of 1,200 Hz and streamed in real-time to the same lab computer as ultrasound.

In both sets of experiments, the 128-element linear array transducer of a portable, handheld ultrasound scanner (mSonics, Lonshine Technologies Inc., Beijing, China) was affixed to the thigh of each subject (on the same limb as surface EMG) via a custom-designed probe holder. The ultrasound transducer was placed on the anterior thigh during the first set of ambulation tasks, and on the posterior thigh during the second set of ambulation tasks. Priority was given to surface EMG to ensure optimal recording from the desired muscles when placing the ultrasound transducer on the anterior and posterior thigh. For anterior thigh ultrasound trials, the transducer was placed transversely at approximately half the distance between the anterior superior iliac spine and the proximal base of the patella (above the surface EMG electrodes) to collect grayscale images of the rectus femoris (RF), vastus medialis (VM) and vastus intermedius (VI) muscles. During the posterior thigh ultrasound trials, the transducer was placed transversely over the belly of the biceps femoris (BF) and semitendinosus muscles (below the surface EMG electrodes). Correct ultrasound placement, transversely over the belly of the desired muscles (Fukunaga et al., 1997), was confirmed visually via grayscale images prior to securing the transducer in both anterior thigh and posterior thigh positions. Ultrasound transmit frequency was set to 7.5 MHz for all trials and the overall image gain was adjusted to optimize the brightness of the image and preserve image resolution at the deep muscle boundary.

Forty-two reflective markers were placed over anatomical landmarks following guidelines by Delagi *et al.* on the bilateral feet, shanks, and thighs, as well as trunk and pelvis for collection of kinematic data via a ten-camera Vicon system (Vicon Motion Systems, Oxford, UK) (Delagi et al., 2011). Kinematic data was recorded to the same lab computer as ultrasound and surface EMG at a sampling rate of 100 Hz (Peng et al., 2016). Following sensor placement, all subjects were asked to complete two sets of five ambulation tasks: level walk, 10° incline walk, 10° decline walk, stair ascent and stair descent. All walking tasks were completed for 1 minute at a self-selected pace on a split-belt treadmill (Bertec, Columbus, OH, United States). Stair trials were completed on a four-step stair case, beginning with stair ascent followed by stair descent, with subjects walking in a reciprocal gait pattern at a self-selected pace. Stair trials were repeated five times and walk-to-stair and stair-to-walk transition strides were included in the respective stair analyses. Hip, knee and ankle kinematic data were calculated in Visual 3D via inverse kinematics, and a custom MATLAB program was created to enable time stamping of data for synchronization of sonomyography and surface EMG with kinematic data.

### Sensor Feature Generation

Raw EMG signals were first processed to remove any baseline offset, then a fourth-order Butterworth bandpass filter was applied with a low cut-off at 20 Hz and a high cut-off at 450 Hz to remove motion artifact and high frequency noise, respectively (De Luca et al., 2010; Chowdhury et al., 2013). Then, a sliding analysis window method was used for extracting six features from processed surface EMG data and has been described in detail previously (Huang et al., 2011; Rabe et al., 2021a). The six features included four time-domain features (mean absolute value, number of slope sign-changes, number of zero-crossings and waveform length), as well as the first and second coefficient of a fourth-order autoregressive model. Sliding windows of length 200 ms were used with a 50 ms overlap, generating a 20 Hz signal of six EMG features from each of the eight muscles, resulting in 48 features per time-point (Smith et al., 2011).

The changes in grayscale ultrasound image intensity are correlated to changes in muscle density, as well as muscle architecture features such as muscle thickness and fascicle dynamics. Many researchers have evaluated these ultrasound-based features of muscle morphology for correlations with muscle force production, muscle contraction, and joint motion as well as overall muscle strength and muscle fatigue (Kurokawa et al., 2001; Muraoka et al., 2005; Blazevich et al., 2006; Han et al., 2013; Panizzolo et al., 2013; Li et al., 2020). Based on previous research demonstrating these features are useful for estimation of knee kinematics from sonomyography, mean intensity and temporal intensity features were extracted from each ultrasound imaging frame, as described in detail previously (Jahanandish et al., 2019a; Jahanandish et al., 2019b; Rabe et al., 2020a; Rabe et al., 2021a; Rabe et al., 2021b). The image sequence from each trial was split by heel strikes to create an ultrasound image sequence for each stride. A spatial filter with a block size of 3 × 3 mm was used to extract mean intensity of each 3 × 3 mm block. Then, this 2-dimensional array of mean image intensity features was rearranged into 1-dimension by horizontally concatenating rows of features ranging from superficial to deep image features. The temporal features were created by taking the time derivative of each feature set between consecutive frames. Finally, the mean intensity and temporal intensity features were combined to create a single sonomyography feature set consisting of 520 features per frame.

### Regression Model

**FIGURE 2:**
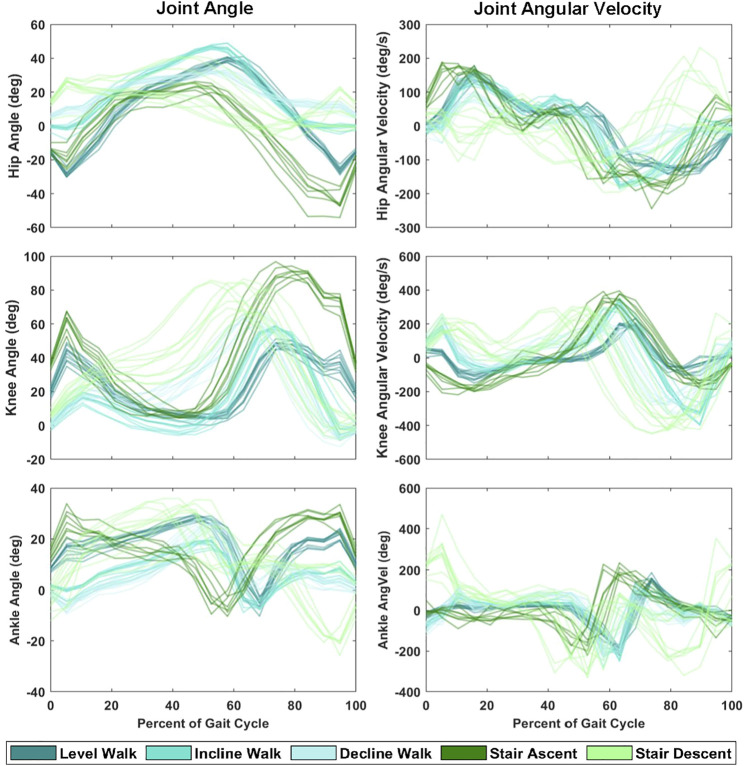
Hip, knee and ankle joint angle and angular velocity during ten strides of each ambulation task (level walk, incline walk, decline walk, stair ascent and stair descent) from a single representative subject.

The Gaussian process regression model is a Bayesian non-parametric kernel-based model that has been introduced as a real-time approximation of inverse dynamics solutions as an alternative for musculoskeletal model-based calculations (Rasmussen, 2004; Nguyen-Tuong et al., 2008; Wang et al., 2008). These models offered accurate function approximation given high-dimensional inputs at a relatively low computational demand to their inverse dynamics counterpart. In this study, we make the assumption that the distribution of joint kinematics within a subject, across multiple strides of a given task (e.g., Figure 2), can be characterized by a Gaussian distribution. Based on these preliminary analyses and previous work, we chose to implement a Gaussian process regression model with a rational quadratic kernel for solving the joint kinematics given the sonomyography, surface EMG, and sensor fusion feature sets. This model has been described in detail previously (Rasmussen, 2004; Zhang et al., 2018), but we assume the general function f(x) is distributed as a Gaussian function with a mean function m(x) and covariance, or kernel, function $k\left(x,x^{\prime }\right)$

$$f\left(x\right)\;∼\mathrm{GP}\left(m\left(x\right),k\left(x,x^{\prime }\right)\right)$$ (1)

The mean function reflects the expected function value at an input, x , and the rational quadratic kernel is described as

$$k\left(x,\mathrm{x\prime}\text{|}\theta \right)=\sigma^{2}\left(1+\frac{r^{2}}{2\alpha l^{2}}\right)$$ (2)

with

$$r=\;\sqrt{{\left(x-\mathrm{x\prime}\right)}^{T}\left(x-\mathrm{x\prime}\right)},$$ (3)

θ is the maximum a posteriori estimates, σ  is the signal standard deviation or amplitude parameter, and α is the non-negative parameter of the covariance or scale mixture, and l is the length-scale (Schulz et al., 2018). A block coordinate descent method was used to solve for model parameters during training with a block size of 1,000 observations, gradient tolerance of 0.001, step tolerance of 0.001 and a maximum of 100,000 iterations to reach convergence.

Each of the three feature sets: sonomyography, surface EMG, and sensor fusion were used as inputs to separate Gaussian process regression models for subject-dependent training and testing. Leave-one-stride-out cross-validation was used to prevent over-fitting of the model. In other words, a pooled dataset containing strides from all ambulation tasks was created and one stride of each ambulation task was removed for testing, then looped through the strides such that each stride of each ambulation task was the test stride once.

### Statistical Analysis

Root mean square errors (RMSE) of each ambulation task and overall root mean square errors across ambulation tasks were calculated, and descriptive statistics (mean and standard deviations (SD)) were reported. A one-way ANOVA was completed to compare RMSE across sensing modalities within ambulation tasks at the hip, knee and ankle joint (*α* = 0.05). When a group significant difference was found, subsequent Tukey-Kramer multiple comparisons tests were completed to determine significant differences between sensing modalities. Additionally, RMSE were normalized to the range of the measured kinematics for comparison across joints and tasks. Additionally, an adjusted coefficient of determination $\left(R^{2}\right)$ for nonlinear regression was calculated as a goodness-of-fit measure for the predicted kinematic trajectories.

Adjusted R2=1−N−1N−1−p(1−R2) (4)

where

$$R^{2}=1-\frac{\mathrm{Residual Sum of Squares}}{\mathrm{Total Sum of Squares}},$$ (5)

N is the size of the training set, and p is the number of variables in the regression model.

## Results

Fusion of features from anterior sonomyography with surface EMG resulted in the lowest overall RMSE averaged across all ambulation tasks at the hip, knee and ankle (Table 2, Table 3, Table 4). At the hip, there was a significant difference in overall average RMSE when comparing hip angles predicted from features of EMG with anterior sonomyography, as well as both anterior and posterior sensor fusion. Additionally, there was a significant difference in overall average RMSE when comparing hip angles from features of posterior sonomyography with anterior sensor fusion. There was a significant difference in overall average RMSE when comparing the hip angular velocities from features of EMG with anterior sensor fusion. At the knee, there was a significant difference in overall knee angle RMSE from features of EMG with all other sensing modalities, in addition to features from posterior sonomyography with anterior sensor fusion. Significant differences were found between overall RMSE of knee angular velocity prediction from features of EMG with anterior sonomyography and anterior sensor fusion. There was an additional significant difference in overall RMSE of knee angular velocity prediction between posterior sonomyography and anterior sensor fusion features. Lastly, at the ankle, there were significant differences between overall RMSE of ankle angle between EMG and anterior sonomyography, anterior sensor fusion and posterior sensor fusion. There was a significant difference between RMSE of ankle angle prediction based on posterior sonomyography features and anterior sensor fusion features. There were no significant differences between RMSE of all sensing modalities prediction of ankle angular velocity.

**TABLE 2:** Mean (SD) root mean square error (RMSE) and range-normalized RMSE (nRMSE) of hip angle and angular velocity during level walking, incline walking, decline walking, stair ascent and stair descent. Joint kinematics were predicted by Gaussian process regression models trained and tested on features from five sensing modalities: 1) surface electromyography (EMG), 2) anterior sonomyography (Ant. SMG), 3) posterior sonomyography (Pos. SMG), 4) sensor fusion of Ant. SMG with EMG (Ant. Fusion), and 5) sensor fusion of Pos. SMG with EMG (Pos. Fusion). Overall average values are mean across all ambulation tasks.

Ambulation task	Mean (SD) hip angle RMSE (deg)					nRMSE hip angle (%)				
	EMG	Ant. SMG	Pos. SMG	Ant. Fusion	Pos. Fusion	EMG	Ant. SMG	Pos. SMG	Ant. Fusion	Pos. Fusion
Level walk	3.18 (1.06)	2.21 (0.51)	3.33 (3.03)	1.63 (0.26)	2.28 (1.01)	8.3%	5.8%	8.2%	4.3%	5.8%
Incline walk	5.55 (2.03)^a^	2.77 (1.65)	2.98 (0.88)	2.10 (0.73)	2.37 (0.55)	10.1%	5.1%	5.5%	3.9%	4.4%
Decline walk	2.98 (0.76)^b^ ^,^ ^c^ ^,^ ^d^	1.77 (0.60)	2.13 (0.70)	1.59 (0.57)	1.82 (0.62)	13.4%	7.6%	9.4%	6.9%	8.0%
Stair ascent	7.65 (5.02)	4.73 (1.15)	6.92 (3.50)	4.05 (1.15)	6.30 (3.40)	15.4%	10.9%	13.0%	9.3%	11.9%
Stair descent	4.90 (1.39)	3.29 (1.18)	5.06 (2.66)	2.76 (1.05)	4.57 (2.01)	20.1%	13.7%	20.0%	11.6%	18.1%
Overall average	4.85 (2.05)^b^ ^,^ ^c^ ^,^ ^d^	2.96 (1.02)	4.08 (2.15)^e^	2.43 (0.75)	3.47 (1.52)	13.5%	8.6%	11.2%	7.2%	9.6%

Ambulation task	Mean (SD) hip angular velocity RMSE (deg/s)					nRMSE hip angular velocity (%)				
	EMG	Ant. SMG	Pos. SMG	Ant. Fusion	Pos. Fusion	EMG	Ant. SMG	Pos. SMG	Ant. Fusion	Pos. Fusion
Level walk	40.32 (10.18)	34.45 (10.87)	35.33 (13.42)	33.20 (10.61)	33.57 (13.33)	17.5%	14.4%	15.3%	14.1%	14.5%
Incline walk	27.56 (4.62)^c^ ^,^ ^d^	19.64 (8.73)	20.12 (5.21)	17.40 (7.05)	16.99 (4.17)	10.8%	7.7%	7.7%	6.8%	6.5%
Decline walk	18.30 (4.43)	14.63 (4.34)	17.54 (4.76)	12.93 (4.06)	14.68 (3.79)	14.0%	11.2%	12.6%	10.0%	10.7%
Stair ascent	41.50 (14.06)	31.06 (5.24)	45.17 (22.44)	28.09 (5.75)	40.45 (21.63)	15.1%	12.7%	14.4%	11.4%	12.9%
Stair descent	33.45 (11.58)	22.65 (5.83)	29.08 (11.46)	21.83 (5.98)	27.47 (11.10)	20.1%	14.0%	17.2%	13.4%	16.2%
Overall average	32.23 (8.97)^c^	24.49 (7.00)	29.45 (11.46)	22.69 (6.69)	26.63 (10.81)	15.5%	12.0%	13.4%	11.1%	12.2%

a Indicates significant difference (*p*< 0.05) between RMSE, of EMG, and all other sensing modalities.

b Indicates significant difference between (*p*< 0.05) RMSE, of EMG, and anterior SMG.

c Indicates significant difference between (*p*< 0.05) RMSE, of EMG, and anterior sensor fusion (anterior SMG, with EMG).

d Indicates significant difference between (*p*< 0.05) RMSE, of EMG, and posterior sensor fusion (posterior SMG, with EMG).

e Indicates significant difference between (*p*< 0.05) RMSE, of posterior SMG, and anterior sensor fusion (anterior SMG, with EMG).

**TABLE 3:** Mean (SD) root mean square error (RMSE) and range-normalized RMSE of knee angle and angular velocity during level walking, incline walking, decline walking, stair ascent and stair descent. Joint kinematics were predicted by Gaussian process regression models trained and tested on features from five sensing modalities: 1) surface electromyography (EMG), 2) anterior sonomyography (Ant. SMG), 3) posterior sonomyography (Pos. SMG), 4) sensor fusion of Ant. SMG with EMG (Ant. Fusion), and 5) sensor fusion of Pos. SMG with EMG (Pos. Fusion). Overall average values are mean across all ambulation tasks.

Ambulation task	Mean (SD) knee angle RMSE (deg)					nRMSE knee angle (%)				
	EMG	Ant. SMG	Pos. SMG	Ant. Fusion	Pos. Fusion	EMG	Ant. SMG	Pos. SMG	Ant. Fusion	Pos. Fusion
Level walk	7.36 (2.08)^b^ ^,^ ^c^ ^,^ ^d^	4.73 (1.25)	5.84 (3.00)	3.77 (0.81)	4.42 (1.85)	11.7%	7.6%	9.2%	6.0%	6.9%
Incline walk	6.62 (0.99)^a^	4.03 (1.39)	4.53 (1.10)	3.22 (0.91)	3.58 (0.94)	13.0%	7.7%	3.9%	6.2%	7.0%
Decline walk	7.37 (2.12)^a^	4.97 (1.10)	5.40 (1.36)	3.94 (1.03)	4.02 (1.03)	11.2%	7.6%	8.3%	6.0%	6.1%
Stair ascent	13.78 (7.21)^b^ ^,^ ^c^	8.56 (1.56)	11.52 (4.75)	7.63 (1.56)	10.92 (4.26)	19.8%	12.7%	16.5%	11.4%	15.4%
Stair descent	14.90 (3.28)^b^ ^,^ ^c^	8.25 (2.95)	12.40 (4.37)^e^	7.67 (2.57)	11.09 (3.74)	17.9%	9.9%	14.9%	9.3%	13.3%
Overall average	10.01 (3.13)^a^	6.11 (1.65)	7.94 (2.92)^e^	5.25 (1.38)	6.80 (2.36)	14.7%	9.1%	11.6%	7.8%	9.8%

Ambulation task	Mean (SD) knee angular velocity RMSE (deg/s)					nRMSE knee angular velocity (%)				
	EMG	Ant. SMG	Pos. SMG	Ant. Fusion	Pos. Fusion	EMG	Ant. SMG	Pos. SMG	Ant. Fusion	Pos. Fusion
Level walk	93.67 (16.54)^b^ ^,^ ^c^	67.33 (22.56)	80.96 (20.74)	64.57 (19.03)	79.50 (15.69)	15.6%	11.3%	13.4%	10.9%	13.2%
Incline walk	51.59 (8.66)^b^ ^,^ ^c^ ^,^ ^d^	35.93 (14.58)	40.98 (6.61)^e^	27.61 (9.13)	35.27 (7.99)	14.4%	9.6%	11.4%	7.4%	9.8%
Decline walk	61.63 (11.82)	53.12 (14.00)	61.70 (18.98)	45.37 (11.22)	48.50 (14.05)	12.4%	10.7%	12.0%	9.1%	9.5%
Stair ascent	90.61 (28.58)^c^	62.77 (10.78)	81.20 (30.33)	55.20 (13.59)	74.55 (28.59)	19.0%	13.0%	16.8%	11.3%	15.8%
Stair descent	111.38 (27.31)^b^ ^,^ ^c^	72.41 (21.90)	104.14 (37.79)	67.12 (16.00)	93.59 (35.41)	18.9%	12.3%	17.7%	11.5%	15.9%
Overall average	81.77 (18.58)^b^ ^,^ ^c^	58.31 (16.76)	73.80 (22.89)^e^	51.97 (13.79)	66.28 (20.35)	16.1%	11.4%	14.3%	10.0%	12.8%

a Indicates significant difference (*p*< 0.05) between RMSE, of EMG, and all other sensing modalities.

b Indicates significant difference between (*p*< 0.05) RMSE, of EMG, and anterior SMG.

c Indicates significant difference between (*p*< 0.05) RMSE, of EMG, and anterior sensor fusion (anterior SMG, with EMG).

d Indicates significant difference between (*p*< 0.05) RMSE, of EMG, and posterior sensor fusion (posterior SMG, with EMG).

e Indicates significant difference between (*p*< 0.05) RMSE, of posterior SMG, and anterior sensor fusion (anterior SMG, with EMG).

**TABLE 4:** Mean (SD) root mean square error (RMSE) and range-normalized RMSE of ankle angle and angular velocity during level walking, incline walking, decline walking, stair ascent and stair descent. Joint kinematics were predicted by Gaussian process regression models trained and tested on features from five sensing modalities: 1) surface electromyography (EMG), 2) anterior sonomyography (Ant. SMG), 3) posterior sonomyography (Pos. SMG), 4) sensor fusion of Ant. SMG with EMG (Ant. Fusion), and 5) sensor fusion of Pos. SMG with EMG (Pos. Fusion). Overall average values are mean across all ambulation tasks.

Ambulation task	Mean (SD) ankle angle RMSE (deg)					nRMSE ankle angle (%)				
	EMG	Ant. SMG	Pos. SMG	Ant. Fusion	Pos. Fusion	EMG	Ant. SMG	Pos. SMG	Ant. Fusion	Pos. Fusion
Level walk	2.99 (0.78)	2.55 (0.57)	2.89 (1.31)	2.21 (0.47)	2.41 (1.04)	12.5%	10.6%	12.0%	9.2%	10.0%
Incline walk	4.51 (0.95)^a^	2.66 (0.91)	3.25 (1.01)	2.29 (0.70)	2.80 (0.91)	18.7%	10.9%	13.2%	9.4%	11.4%
Decline walk	3.81 (1.20)^a^	2.34 (0.68)	2.61 (0.30)	2.17 (0.55)	2.15 (0.44)	14.1%	8.4%	9.6%	7.8%	7.9%
Stair ascent	6.18 (3.44)	4.36 (1.27)	6.30 (2.60)	3.95 (1.54)	5.17 (1.81)	20.6%	14.1%	20.6%	12.2%	18.2%
Stair aescent	7.93 (2.28)^b^ ^,^ ^c^	4.83 (1.75)	6.52 (2.30)	4.36 (1.54)	5.66 (2.10)	16.3%	9.7%	13.2%	8.5%	12.2%
Overall average	5.08 (1.73)^b^ ^,^ ^c^ ^,^ ^d^	3.35 (1.04)	4.31 (1.51)^e^	3.00 (0.96)	3.64 (1.26)	16.4%	10.8%	13.7%	9.4%	11.9%

Ambulation task	Mean (SD) ankle angular velocity RMSE (deg/s)					nRMSE ankle angular velocity (%)				
	EMG	Ant. SMG	Pos. SMG	Ant. Fusion	Pos. Fusion	EMG	Ant. SMG	Pos. SMG	Ant. Fusion	Pos. Fusion
Level walk	47.12 (13.40)	45.90 (10.30)	48.29 (17.21)	41.36 (8.11)	44.76 (16.15)	16.3%	16.2%	16.5%	14.8%	15.2%
Incline walk	43.88 (11.35)	36.64 (10.11)	43.80 (14.15)	33.32 (8.07)	40.39 (13.72)	15.2%	13.7%	16.0%	12.4%	14.8%
Decline walk	43.29 (7.95)	42.52 (7.91)	44.36 (6.16)	40.14 (7.59)	41.66 (5.50)	18.1%	17.9%	18.5%	16.9%	17.3%
Stair ascent	62.59 (25.86)	50.19 (10.78)	67.81 (32.65)	46.83 (9.93)	66.20 (33.24)	21.8%	19.4%	22.2%	18.2%	21.6%
Stair descent	82.44 (18.49)	67.31 (14.37)	78.38 (21.28)	65.25 (10.65)	75.55 (21.92)	18.4%	15.7%	17.0%	15.1%	16.3%
Overall average	55.87 (15.41)	48.51 (10.70)	56.53 (18.29)	45.48 (8.87)	53.71 (18.11)	18.2%	16.6%	18.0%	15.5%	17.1%

a Indicates significant difference (*p*< 0.05) between RMSE, of EMG, and all other sensing modalities.

b Indicates significant difference between (*p*< 0.05) RMSE, of EMG, and anterior SMG.

c Indicates significant difference between (*p*< 0.05) RMSE, of EMG, and anterior sensor fusion (anterior SMG, with EMG).

d Indicates significant difference between (*p*< 0.05) RMSE, of EMG, and posterior sensor fusion (posterior SMG, with EMG).

e Indicates significant difference between (*p*< 0.05) RMSE, of posterior SMG, and anterior sensor fusion (anterior SMG, with EMG).

**FIGURE 3:**
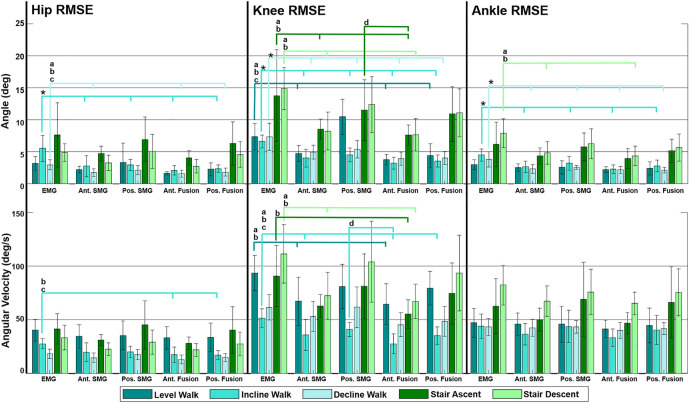
Mean of all subjects (*N* = 9) root mean square error (RMSE) of hip, knee and ankle angle and angular velocity predicted by Gaussian process regression models trained by multiple feature sets during five ambulation tasks. Feature sets include surface electromyography (EMG), anterior sonomyography (Ant. SMG), posterior sonomyography (Pos. SMG), Ant. SMG sensor fusion (Ant. Fusion), and Pos. SMG sensor fusion (Pos. Fusion). RMSEs were calculated between sensor-based prediction of joint kinematics and estimated kinematics. Error bars display standard deviations for the respective RMSE. Significance bars indicate significant difference (*p* < 0.05) between RMSE of (*) EMG and all other sensing modalities, (a) EMG and anterior SMG, (b) EMG and anterior sensor fusion, (c) EMG and posterior sensor fusion (posterior SMG with EMG), and (d) posterior SMG and anterior sensor fusion (anterior SMG with EMG).

Comparing across joints, the lowest RMSEs for both angle and angular velocity are observed at the hip and ankle, where the overall magnitude of the joint angle and angular velocity are lower (Figure 3). The normalized RMSE revealed that the hip angle and angular velocity were the lowest, there was an increase in normalized RMSE of knee angle and angular velocity, and the greatest normalized RMSE was observed when predicting ankle angle and angular velocity across most sensing modalities. Within the hip, knee and ankle joints individually, the normalized RMSE reveals that there was increased difficulty predicting joint angular velocity in comparison to joint angle for all sensing modalities.

### Hip Estimation Performance

**FIGURE 4:**
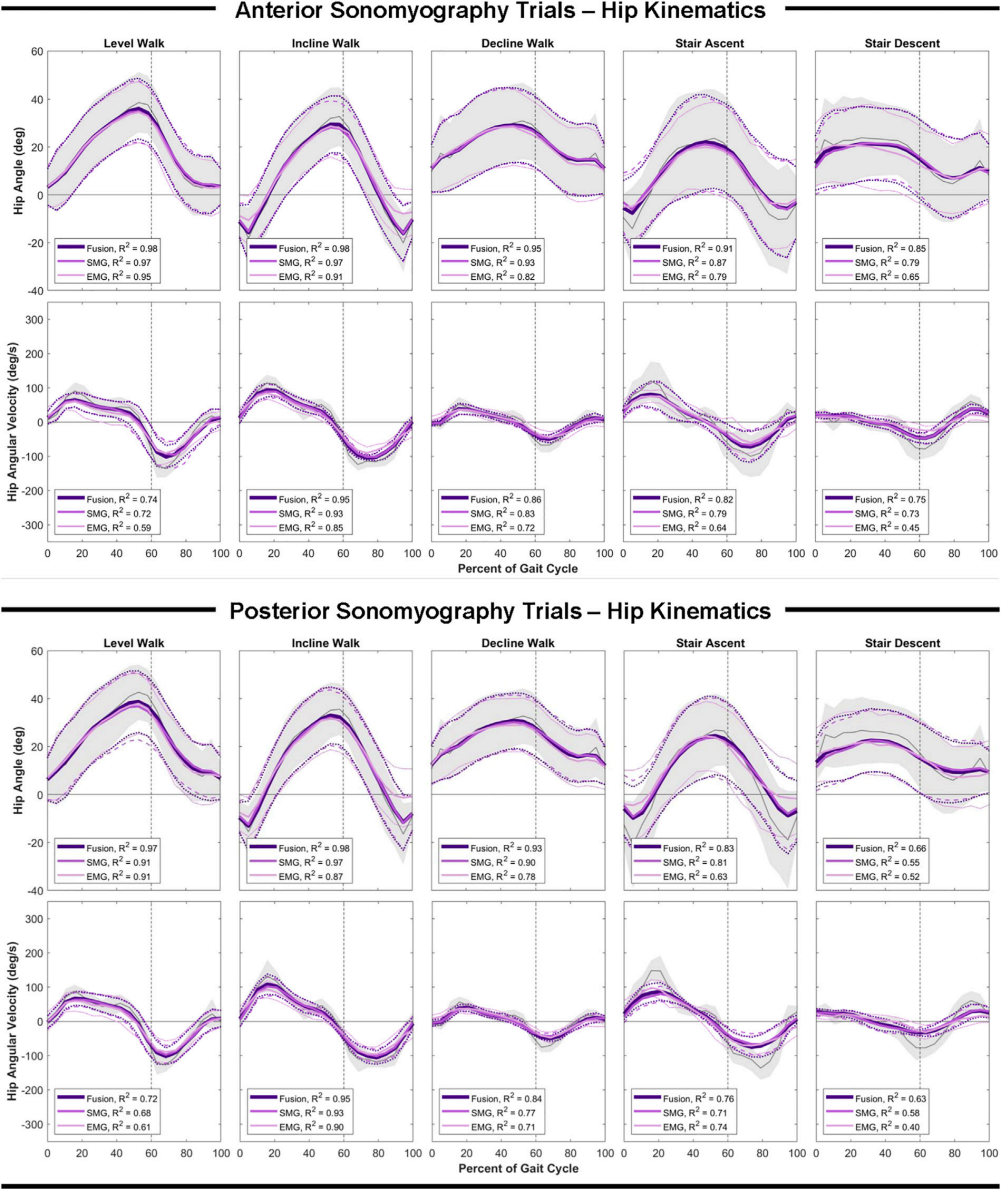
Hip angle and angular velocity as a function of the gait cycle. Measured kinematics are displayed in gray with standard deviations in shaded regions. Predicted kinematics from Gaussian process regression models trained and tested on features from electromyography (EMG), sonomyography (SMG) and sensor fusion (Fusion) are displayed with respective standard deviations. Adjusted R^2^ given as a goodness-of-fit metric for each sensing modality compared to the measured kinematics.

Anterior sensor fusion resulted in the best predictive performance of the Gaussian process regression model for both hip angle and angular velocity during all ambulation tasks, as evidenced by RMSE, normalized RMSE and the adjusted R^2^ values (Table 2; Figure 4). There were no significant differences in RMSE of hip angle and angular velocity between anterior sonomyography alone and anterior sensor fusion, although anterior sensor fusion consistently outperformed anterior sonomyography. Surface EMG resulted in the worst predictive performance for all walking and stair tasks, followed by posterior sonomyography. The regression model predicted hip angle and angular velocity best during incline walking strides, followed by level walking strides for all sensing modalities. The lowest performance (greatest RMSE and normalized RMSE, lowest adjusted R^2^) of regression model prediction of hip angle and angular velocity was observed during stair strides for all sensing modalities, with stair descent resulting in the greatest error.

### Knee Estimation Performance

**FIGURE 5:**
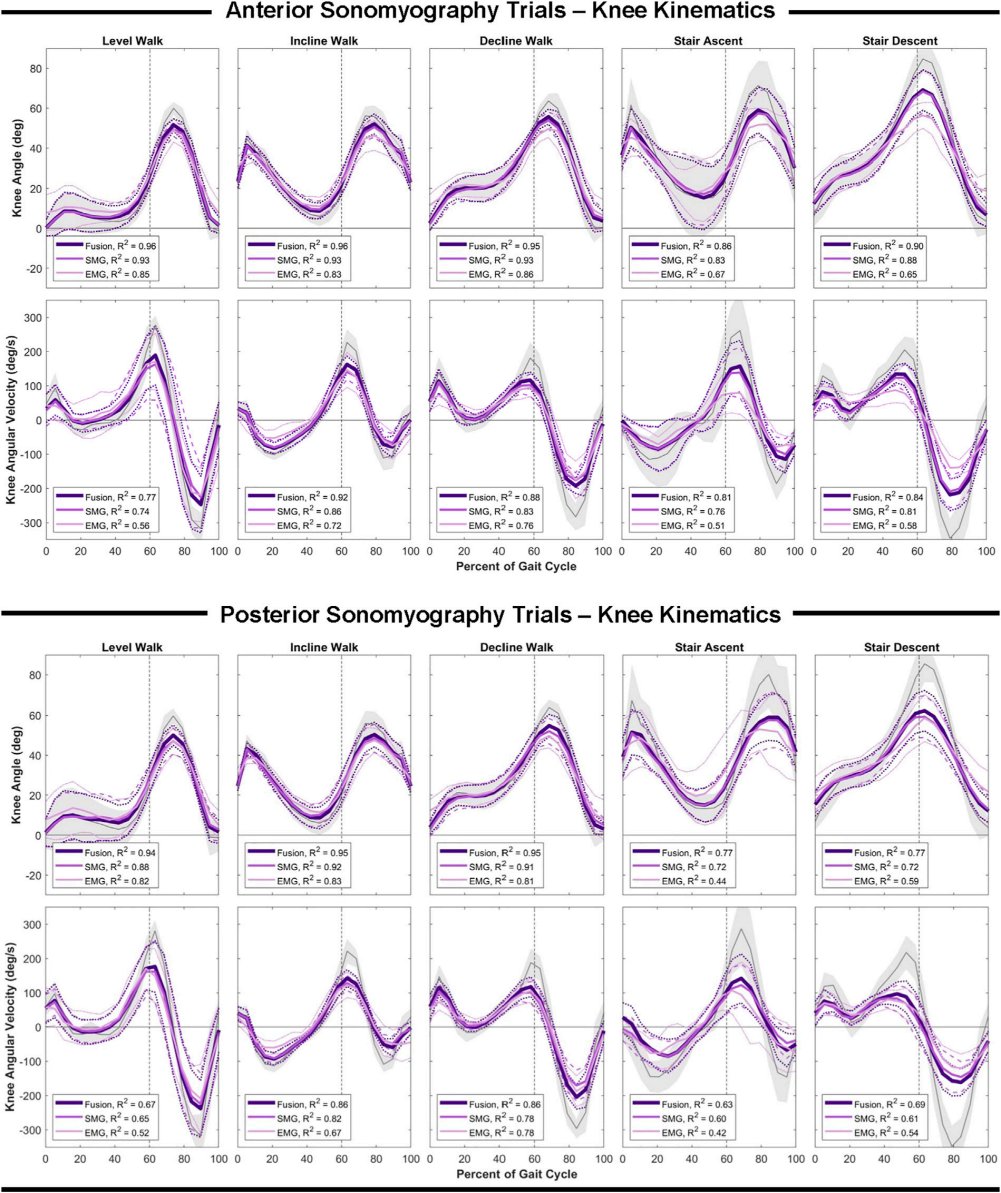
Knee angle and angular velocity as a function of the gait cycle. Measured kinematics are displayed in gray with standard deviations in shaded regions. Predicted kinematics from Gaussian process regression models trained and tested on features from electromyography (EMG), sonomyography (SMG) and sensor fusion (Fusion) are displayed with respective standard deviations. Adjusted R^2^ given as a goodness-of-fit metric for each sensing modality compared to the measured kinematics.

Similar to the hip, anterior sensor fusion resulted in the best predictive performance of the Gaussian process regression model for both knee angle and angular velocity during all ambulation tasks (Table 3; Figure 5). There were no significant differences in RMSE of knee angle and angular velocity between anterior sonomyography alone and anterior sensor fusion. Surface EMG resulted in the worst predictive performance for all ambulation tasks, followed by posterior sonomyography. The regression model predicted knee angle during level and incline walking with the lowest error for all sensing modalities compared to decline walking and stair strides. The lowest error for knee angular velocity prediction was observed during incline walking for regression models trained from all sensing modalities, followed by decline walking, level walking, and stair strides, as evidenced by the normalized RMSE and adjusted R^2^ values.

### Ankle Estimation Performance

**FIGURE 6:**
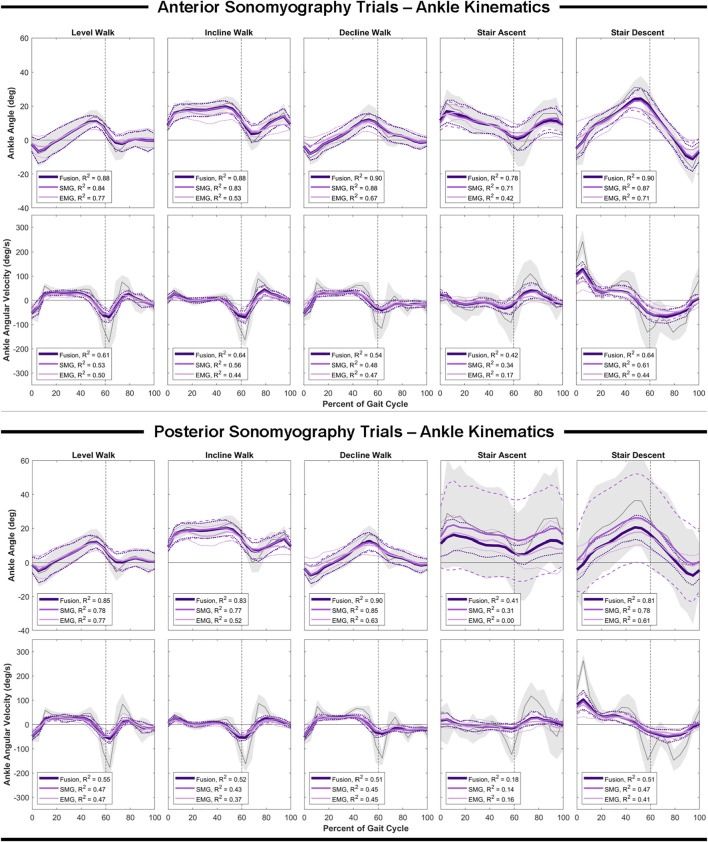
Ankle angle and angular velocity as a function of the gait cycle. Measured kinematics are displayed in gray with standard deviations in shaded regions. Predicted kinematics from Gaussian process regression models trained and tested on features from electromyography (EMG), sonomyography (SMG) and sensor fusion (Fusion) are displayed with respective standard deviations. Adjusted R^2^ given as a goodness-of-fit metric for each sensing modality compared to the measured kinematics.

In comparison to the hip and knee, the Gaussian process regression model performed worst at the ankle for all sensing modalities. However, comparing sensing modalities, the results remain consistent that anterior sensor fusion resulted in the lowest error of ankle angle and angular velocity, followed by anterior sonomyography alone (Table 4; Figure 6). Surface EMG resulted in the greatest error for ankle angle prediction during all ambulation tasks, with significant improvement between all other sensing modalities versus EMG during incline and decline walking. For both anterior sensor fusion and anterior sonomyography-based estimates of ankle angle, the highest performance (lowest normalized RMSE and greatest adjusted R^2^) was observed during decline walking, followed by stair descent, incline walking, level walking, and lastly, stair ascent. There were no significant differences between RMSE of ankle angular velocity prediction by any sensing modality during any of the five ambulation tasks.

### Computational Expense

All models were trained and tested offline on a single CPU (Intel(R) Core i7-7700 at 3.60 GHz). Average time for training and testing a single fold of the leave-one-stride-out framework for the rational quadratic Gaussian process regression model are given in Table 5.

**TABLE 5:** Mean (SD) computational time to train the Gaussian process regression model using three separate feature sets containing strides from all five ambulation tasks and test on individual strides of each ambulation task for the hip, knee and ankle.

Feature set	Training time (s)	Testing time (ms)
Surface electromyography	6.48	1.3
Anterior sonomyography	6.22	4.4
Posterior sonomyography	5.93	4.7
Anterior sensor fusion	23.45	5.4
Posterior sensor fusion	17.89	5.4

## Discussion

The objective of this research was to evaluate features of surface EMG and sonomyography, as well as fusion of these features, for the continuous prediction of hip, knee and ankle angle and angular velocity. In support of our hypothesis, sonomyography features and sensor fusion of sonomyography with surface EMG, consistently resulted in the greatest predictive performance for hip, knee and ankle angles and angular velocities in comparison to surface EMG alone. However, for all joints (hip, knee *and* ankle), there were no significant differences between RMSE of angle and angular velocity prediction based on the respective anterior or posterior sonomyography and sensor fusion features, and anterior sonomyography and sensor fusion resulted in relatively lower error compared to posterior sonomyography and sensor fusion. These results are surprising at the ankle, where the addition of information of muscle activity from muscles that span that joint did not significantly improve the regression performance. The increased performance, or reduced error, of anterior sonomyography in comparison to posterior sonomyography, could possibly be attributed to capturing features from three muscles on the anterior thigh (rectus femoris, vastus intermedius, and vastus medialis), as opposed to only two muscles on the posterior thigh (biceps femoris and semitendinosus). This gives further justification to the increased resolution, as well as ability to access deep muscle tissue, as probable explanations for the improved regression performance of sonomyography in comparison to surface EMG. These results are in agreement with our previous work demonstrating increased performance of sonomyography in comparison to surface EMG for ambulation mode classification, as well as high performance of sonomyography-based knee angular velocity prediction and hip, knee and ankle joint moment prediction (Rabe et al., 2020a; Rabe et al., 2021a; Rabe et al., 2021b).

For all sensing modalities, model error increased when comparing prediction of joint angle and angular velocity during walking tasks to stair ambulation tasks. This can likely be explained by the decrease in the number of stair strides in the training dataset compared to the level, incline and decline walking strides, or perhaps the increased variability of the stair ambulation kinematic trajectories. Another possible explanation is the need for additional high resolution (i.e., sonomyography) information from additional thigh muscles during the stair ambulation tasks to account for the increased variability during these tasks. Previous research showed that muscle activity of the individual quadriceps and hamstrings muscles is reduced during stair ascent and stair descent compared to level walking in healthy individuals; however, there is an opposite relationship in muscle coactivity where there is an increase in hamstrings/quadriceps coactivity during stair ambulation as opposed to level walking (Bae et al., 2009). These results point to the potential benefit of sonomyography from the anterior thigh muscles and posterior thigh muscles simultaneously to improve the prediction of hip, knee and ankle joint kinematics.

Accurate prediction of joint output kinematics from a reliable sensing modality is vital to the success of powered assistive devices. While many researchers evaluate control over multiple degrees-of-freedom in the upper-limb, research evaluating simultaneous control strategies over the hip, knee and ankle degrees-of-freedom of the lower limb is less widely reported. The first powered lower-limb assistive devices focused only on powering a single joint, such as the knee or ankle alone (Au et al., 2007; Fite et al., 2007). However, the technological advancement of powered lower-limb assistive devices has enabled the plausibility for multiple powered joints within a single device. Minimizing the complexity of the control strategy for these devices will expediate their entrance into a rehabilitation clinic setting and the hands of users. To date, researchers have relied on pre-programmed trajectories for various ambulatory tasks, such as stair ascent or ramp walking (Lawson et al., 2013; Simon et al., 2014; Lenzi et al., 2018). These strategies have proven to be successful, but require users to adapt their mechanics to accommodate the device, rather than allowing the device mechanics to adapt to the user. This can result in further injury or long-term wear to the unaffected joints (Grabowski and D’Andrea, 2013). The results from the present study are promising for continuous prediction of hip, knee and ankle kinematics that may be able to adapt to the user.

The Gaussian process regression model was chosen based on its recent success for use as a real-time approximation of inverse dynamics solutions to musculoskeletal model-based control. We believe the Gaussian process regression model is an appropriate model for the non-linear relationship between the sonomyography and surface EMG features from multiple muscles with the output of the hip, knee and ankle joints. As an alternative to this approach, Zhang *et al.* evaluated various ultrasound features of tibialis anterior muscle contraction and surface EMG in a musculoskeletal model-based algorithm for continuous estimation of isometric ankle dorsiflexion moment and compared this model to linear regression as well as a neural network approach (Zhang et al., 2021). While the model-based approach has many advantages, including establishing a functional, “white-box” relationship between the muscle features with the desired output, the results demonstrated there was no significant difference between the musculoskeletal model-based approach and two regression approaches for ankle moment estimation. However, these results indicated that surface EMG features, muscle fascicle length and muscle pennation angle resulted in higher accuracy of isometric ankle moment prediction in comparison to overall mean image echogenicity (of longitudinal images along the tibialis anterior muscle). In a previous study, Zhang *et al.* combined surface EMG muscle activation features with tibialis anterior pennation angle from ultrasound as inputs to a Hill-type neuromusculoskeletal model for prediction of isometric ankle dorsiflexion moment and found that the fusion of the two sensor data improved prediction accuracy in comparison to sole surface EMG or ultrasound alone (Zhang et al., 2020). Additionally, researchers evaluating contributions of sonomyography and electromyography in both the lower- and upper-limb, have found that both signals contribute to accurate prediction of joint and muscle mechanics (Shi et al., 2007; Ruiz-Muñoz and Cuesta-Vargas, 2014; Boyd and Liu, 2020). The present results further support fusion of sonomyography and surface EMG as an input to regression-based approaches for continuous prediction of joint-level output.

The predictive performance of the Gaussian process regression model was greatest when trained on sensor fusion features from anterior sonomyography and surface EMG. For almost all ambulation modes of all joints, anterior sensor fusion resulted in significantly improved hip, knee and ankle angel, as well as hip and knee angular velocity prediction in comparison to surface EMG. There were fewer instances of significant improvement of the prediction of hip, knee and ankle kinematics when comparing anterior sonomyography alone in comparison to surface EMG. However, there was no significant differences between the anterior sonomyography alone and the anterior sensor fusion-based predictions of hip, knee and ankle kinematics. Importantly, the lack of significant differences between sonomyography alone and sensor fusion of sonomyography with surface EMG indicates that the addition of separate features from surface EMG may not greatly improve the performance of the regression model at the hip, knee or ankle joints. This may be evident when considering the more traditional measurement of surface EMG we used in this study, and not true when considering other newer strategies for recording and decomposition of the surface EMG signal (Stango et al., 2015) Additional work is required to further evaluate the comparison of sensor fusion with sonomyography alone to determine if the burden of collecting data from a separate sensing modality is required for accurate prediction of lower-limb kinematics.

All sonomyography and surface EMG data were recorded in real-time and synchronized to the joint kinematics during each ambulation task. However, feature generation and Gaussian process regression model training and testing was completed offline. The average time to train the regression model on the anterior sonomyography fusion dataset was 23.45 s, and the average time to test the models on strides from the hip, knee and ankle was 5.4 ms. The prediction time is within the optimal reported controller delay window of 100–125 ms for myoelectric prostheses (Farrell and Weir, 2007), as well as faster than the update rates of common lower-limb device controllers (Fluit et al., 2020). However, future work is required to determine if the hip, knee and ankle angle and angular velocity prediction times and accuracies are maintained during online implementation. Given previous success in the upper-limb for online sonomyographic control of dexterous hands (Yang et al., 2020), along with an established correlation between online and offline accuracy of a powered prostheses (Hargrove et al., 2015; Lu et al., 2017), we expect the accuracy of the offline joint kinematic prediction to be comparable, and at a minimum correlate with future online results.

Limitations of the present work exist beyond the offline implementation of the regression models. The subject population included only healthy individuals, without any mobility disorders or limb loss. Additional work is required to determine how the sonomyography features will translate to individuals with gait impairments as well as limb loss. Additionally, the ability of these features and the Gaussian process regression model to predict kinematics of unknown ambulation tasks has not been addressed. Future work evaluating the ability of these features to predict hip, knee and ankle kinematics during unknown tasks will improve understanding of their feasibility for implementation in various activities of daily living, such as stepping over an obstacle. Furthermore, alternative features, such as muscle cross-sectional area and aspect ratio (Guo et al., 2010), as well as alternative models, such as more complex neural networks or musculoskeletal models, may improve performance of the surface EMG and sonomyography features for predicting kinematics of unknown (or “untrained”) tasks. To aid in the evaluation of online prediction of joint kinematics from surface EMG and sonomyographic features of muscle contraction of various subject populations including individuals with mobility disorders, steps should be taken to integrate the sensors into the assistive devices and their control schemes. Researchers have begun to develop electrodes for simultaneous surface EMG and grayscale ultrasound recording within a single system at the same muscle position. (Botter et al., 2019; Yeon et al., 2021). Furthermore, a reduction in the number of ultrasound elements, and perhaps a reduction to single amplitude ultrasound elements, could be possible. Sikdar *et al.* has demonstrated success in predicting dexterous finger movements with a single element ultrasound transducer in the upper limb (Sikdar et al., 2014), and sparsity analyses of grayscale ultrasound indicated a reduction to equally spaced single scanlines did not introduce significant error (Akhlaghi et al., 2020). Additionally, the transition to single element amplitude ultrasound sensors could reduce the ambiguity of the transfer function between amplitude signals and grayscale images provided by brightness modulated ultrasound. Theoretically, this will support improvement of the evaluation of task- and user-independent prediction from sonomyography.

This work evaluated a task-invariant approach for continuous prediction of hip, knee and ankle angle and angular velocity during five common, yet widely-varying, ambulation tasks using Gaussian process regression models trained and tested on features from surface EMG, sonomyography, and sensor fusion. Anterior sonomyography sensor fusion with surface EMG significantly improved almost all joint kinematics in comparison to surface EMG alone, while anterior sonomyography alone significantly improved only some of the joint kinematics (mostly at the hip and knee) in comparison to surface EMG alone. However, there were no significant differences between sonomyography alone and sensor fusion. Additionally, the results revealed that anterior sonomyography and anterior sonomyography sensor fusion gives more accurate predictive performance in comparison to posterior sonomyography and posterior sonomyography sensor fusion, respectively. Sensor fusion and the Gaussian process regression models predicted level ground and ramp ambulation kinematics with greater accuracy in comparison to stair kinematics. Larger training datasets including additional stair strides, or perhaps sonomyography data from muscles on the anterior and posterior thigh simultaneously could possibly improve stair kinematic prediction. We believe this able-bodied study is a fundamental contribution to the evaluation and potential integration of sonomyography sensors into powered assistive devices. Improved control over multiple degrees-of-freedom of powered assistive devices is critical for translation of these devices into the daily lives of users and improving the quality of life for individuals with gait impairments or limb loss.

## Data Availability

The raw data supporting the conclusion of this article will be made available by the authors, without undue reservation.
